# Antigen presenting epithelial cells play a pivotal role in airway allergy

**DOI:** 10.1186/2045-7022-5-S4-O8

**Published:** 2015-06-26

**Authors:** Julia Arebro, Lotta Tengroth, Susanna Kumlien Georén, Ola Winqvist, Lars-Olaf Cardell

**Affiliations:** 1 Karolinska Institutet, ENT-department, Stockholm, Sweden; 2 Karolinska Institutet, Dep. of Medicine, Unit of Translational Immunology, Stockholm, Sweden

## Background

Epithelial cells are known to express MHC class II, but their ability to process and present allergens thus contributing to the development of upper airway disease is unclear. The aim of this study was to establish antigen presenting nasal epithelial cells as a significant factor in the allergic reaction.

## Method

Mucosal specimen from human and mice were used to evaluate the ability of nasal epithelial cells to take up antigen, express MHC class II and co-stimulatory molecules and to stimulate antigen-specific activation and proliferation of CD4+ T cells.

## Results

Human nasal epithelial cells were shown to take up dextran, which ended up in intracellular endosome-like structures. In addition, MHC class II and co-stimulatory molecules were found on human and mouse nasal epithelial cells. Functionally, nasal epithelial cells from ovalbumin-sensitized mice activated and induced antigen-specific proliferation of naïve OT-II CD4+ T cells in vitro. A similar activation was not seen in naïve MNECs. Finally, nasal epithelial cells from allergic rhinitis patients were able to activate autologous T cells against Bet v 1 and induce IL-13 release.

## Conclusion

The present data reveal a significant role for airway epithelial cells in antigen presentation by demonstrating their ability to take up, process and present antigens in a class II dependent manner for the activation of allergen-specific CD4+ T cells. Therefore, the present study establish for the first time nasal epithelial cells as important antigen presenting cells in airway allergy by initiating a local adaptive immune response.

